# Therapeutic effects of Qian-Yu decoction and its three extracts on carrageenan-induced chronic prostatitis/chronic pelvic pain syndrome in rats

**DOI:** 10.1186/s12906-016-1553-7

**Published:** 2017-01-25

**Authors:** Keda Zhang, Xiaobin Zeng, Yonggang Chen, Rong Zhao, Hui Wang, Jinhu Wu

**Affiliations:** 1 Department of Pharmacy, Wuhan Third Hospital, Wuhan, People’s Republic of China; 2 Central Laboratory of Longhua Branch, Shenzhen People’s Hospital, Second Clinical Medical College, Jinan University, Shenzhen, People’s Republic of China; 3 Integrated Chinese and Western Medicine Postdoctoral Research Station, Jinan University, Guangzhou, People’s Republic of China; 4 Key Lab for New Drug Research of TCM and Shenzhen Branch, State R&D Centre for Viro-Biotech, Research Institute of Tsinghua University in Shenzhen, Shenzhen, People’s Republic of China; 5 Clinical Laboratory, Wuhan Third Hospital, Wuhan, People’s Republic of China; 6 Department of Pathology, Wuhan Third Hospital, Wuhan, People’s Republic of China

**Keywords:** Qian-Yu decoction, Chronic prostatitis/chronic pelvic pain syndrome, Anti-inflammatory, Polysaccharide, Flavonoid, Saponin, Carrageenan

## Abstract

**Background:**

Qian-Yu decoction (QYD) is a traditional Chinese medicinal recipe composed of *Radix astragali* (*Astragalus membranaceus* (Fisch.) Bunge var. *mongholicus* (Bunge) P.K. Hsiao, Fabaceae), *Herba epimedii* (*Epimedium brevicornum* Maxim., Berberidaceae), *Herba leonuri* (*Leonurus japonicus* Houtt., Lamiaceae), *Cortex phellodendri* (*Phellodendron chinense* Schneid., Rutaceae) and *Radix achyranthis bidentatae* (*Achyranthes bidentata* Bl., Amaranthaceae). This study aimed to evaluate the therapeutic activity of QYD against carrageenan-induced chronic prostatic/chronic pelvic pain syndrome (CP/CPPS) in rats and further elucidate its effective components.

**Methods:**

Three types of components, total polysaccharides, total flavonoids and total saponins were separately extracted from QYD. Carrageenan-induced CP/CPPS rats were intragastrically administered with lyophilized product of QYD, individual extracts and all the combined forms of extracts for three weeks. Prostatic index (PI) was determined and histopathological analysis was performed. The levels of tumor necrosis factor alpha (TNF-α), interleukin-1 beta (IL-1β), cyclooxygenase-2 (COX-2) and prostaglandin E2 (PEG2) in rat prostate tissues were measured using ELISA. The production of inducible nitric oxide synthase (iNOS) was evaluated by an enzymatic activity assay, and the release of nitric oxide (NO) was determined by a nitrate/nitrite assay.

**Results:**

Treatment with QYD significantly ameliorated the histological changes of CP/CPPS rats and reduced the PI by 44.3%, with a marked downregulation of TNF-α (42.8% reduction), IL-1β (45.3%), COX-2 (36.6%), PGE2 (44.2%), iNOS (54.1%) and NO (46.0%). Each of three extracts attenuated the symptom of CP/CPPS, but much more weakly than QYD. The combined administration of three extracts showed efficacy comparable to that of QYD while better than that of any combination of two extracts. A principal component analysis of the six inflammatory mediators as variables indicated that the effects of TS on CP/CPPS were rather different from those of TF and TP, which were similar.

**Conclusions:**

QYD can be beneficial in prevention and treatment of CP/CPPS. Polysaccharides, flavonoids and saponins, as the major effective components of QYD, exert a cooperative effect on CP/CPPS.

## Background

Chronic prostatitis/chronic pelvic pain syndrome (CP/CPPS), characterized by long-term pelvic or perineal pain without evidence of urinary tract infection, accounts for 90-95% of prostatitis cases [1]. As category III prostatitis classified by the National Institute of Health, CP/CPPS has been subdivided into inflammatory (category IIIa) and non-inflammatory (category IIIb) forms, depending on whether white blood cells can be found in semen, post-prostatic massage urine and expressed prostatic secretion of patients [2]. However, recent studies have questioned the differentiation between categories IIIa and IIIb, since both categories have increases in markers of inflammation, such as tumor necrosis factor alpha (TNF-α), interleukin-1beta (IL-1β), nitric oxide (NO), cyclooxygenase-2 (COX-2) and prostaglandin E2 (PGE2) [3–7]. CP/CPPS is now thought to result from an interplay between psychological factors and dysfunctions in the immune, neurological and endocrine systems [8]. Yet still, the exact cause is unknown, and treating is difficult.

The conventional treatments for CP/CPPS are antibiotics, alpha blockers, anti-inflammatory medications and muscle relaxants, often combined with psychological and physical therapies [9]. However, they focus on relieving symptoms rather than curing the condition, and are often found to provide little or only temporary symptom relief. Successful management of CP/CPPS has always been a challenge for both urologists and patients. Phytotherapy has gained increasing popularity with CP/CPPS patients around the world, due to unique mechanisms of action, very few side effects, low cost and high patient acceptance. Two phytotherapeutic agents, quercetin (a natural flavonoid) and cernilton (pollen extracts), have been valued as effective treatments on the basis of clinical evidences [10–12].

Qian-Yu decoction (QYD), a traditional herbal recipe consisting of (*Radix astragali Astragalus membranaceus*. (Fisch.) Bunge var. *mongholicus* (Bunge) P.K. Hsiao, Fabaceae), *Herba epimedii* (*Epimedium brevicornum* Maxim., Berberidaceae), *Herba leonuri* (*Leonurus japonicus* Houtt., Lamiaceae), *Cortex phellodendri* (*Phellodendron chinense* Schneid., Rutaceae) and *Radix achyranthis bidentatae* (*Achyranthes bidentata* Bl., Amaranthaceae), was prescribed for treating CP/CPPS according to the clinical experience of traditional Chinese medicine (see Table 1). Our previous work pointed out that QYD had efficacy in relieving inflammation and pain, promoting blood circulation to remove stasis and increasing immunity in rat models [13]. Many in vitro and in vivo studies have reported that each herbal ingredient of QYD possesses anti-inflammatory effects [14–19]. But, it remains unclear how effective QYD treatment is for CP/CPPS.

**Table 1:** Composition of Qian-Yu decoction

Herbal ingredients	Chinese names	Percentage (%)
*Radix astragali*	Huangqi (黄芪)	40
*Herba epimedii*	Yinyanghuo (淫羊藿)	20
*Herba leonuri*	Yimucao (益母草)	20
*Cortex phellodendri*	Huangbo (黄柏)	10
*Radix achyranthis bidentatae*	Niuxi (牛膝)	10

Therefore, the aim of this study is to evaluate the therapeutic activity of QYD on CP/CPPS and elucidate its effective components. To this end, three types of chemical components, total polysaccharides (TP), total flavonoids (TF) and total saponins (TS), were extracted from QYD. The effects of QYD and its extracts on prostatic index (PI), histological change and production of some inflammatory markers (TNF-α, IL-1β, COX-2, PGE2, inducible nitric oxide synthase (iNOS) and NO) in carrageenan-induced CP/CPPS rats were investigated. Note that the extract types were defined according to the most studied active components of the herbal ingredients. Since the content of total alkaloids in QYD had been found to be extremely low, which might be attributed to their insolubility and instability during decocting [20], alkaloids were not taken into consideration here.

## Methods

### Plant materials and chemicals

Dried herbal materials *Radix astragali*, *Herba epimedii*, *Herba leonuri*, *Cortex phellodendri* and *Radix achyranthes* were purchased from SinoPharm Group (Wuhan, China) and identified by Prof. Yonggang Chen, one of the coauthors. Their voucher specimens have been deposited at the herbarium of the Department of Pharmacy in Wuhan Third Hospital. All chemical reagents were purchased from SinoPharm Chemical Reagent (Shanghai, China), and of highest available purity.

### Preparation of Qian-Yu decoction

Dry herbal materials (100 g) of QYD were weighed out and put into a regular stainless steel pot. One liter water was added to cover the herbs completely. After overnight soaking, the herbs were brought to a rolling boil, and then simmered for 2 h. The liquid was strained and the herbs were kept in the pot for the second cook. One liter water was added, boiled out, and then kept simmering for 2 h again. The liquid was strained and added to that from the initial cook. The combined liquid was concentrated to produce the QYD (100 ml). The lyophilized product (DLP) of the QYD weighed 28.20 g. The TP, TF and TS contents of DLP were determined using the methods as described below.

### HPLC analysis

Chromatographic fingerprint analysis was performed in a Dionex UltiMate 3000 HPLC system (Thermo Scientific, Germering, Germany) equipped with a dioxide array detector (DAD). The separation was conducted on a Hypersil Gold C18 column (4.6 mm × 250 mm, 5.0 μm; Thermo Scientific, Waltham, MA, USA) at 25 °C. The mobile phase was composed of (A) 0.1% formic acid-water solution and (B) methanol in a gradient program: 8.0-40.0% B, 0–15 min; 40.0-65.0% B, 15–35 min; 65.0-92.0% B, 35–45 min; 92.0% B, 45–55 min. The flow rate was set at 1.00 ml/min and the injection volume was 20 μl. The detection wavelength of 254 nm was used.

### Extraction of total polysaccharides

**Fig. 1:**
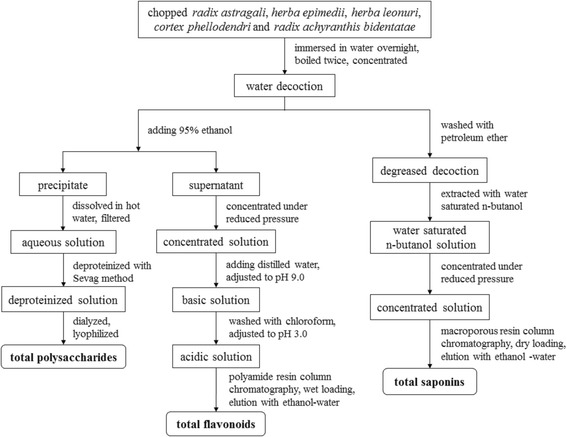
Extraction procedures of total polysaccharides, total flavonoids and total saponins from QYD

Qian-Yu decoction was mixed with 95% ethanol to an ethanol concentration of 80%. The mixture was kept at room temperature overnight, and filtered to collect the precipitated crude polysaccharides and the supernatant from which flavonoids were further separated. The crude polysaccharides were added to hot water, and the undissolved part was removed by filtering. After cooling, the solution was deproteinized using the Sevag reagent (CHCl_3_: n-butanol = 4:1, v/v) [21]. The resulting solution was purified via dialysis (COMW = 14,000 Da) for 18 h, and subjected to lyophilization to produce the final TP extract (Fig. 1).

The content of TP in the extract was determined by the phenol-sulfuric acid colorimetric method using glucose (Must Biotechnology, Chengdu, China) as the standard [22]. Briefly, aqueous solution of glucose (1.00 mg/ml) was diluted to a series of concentrations (0.01-0.05 mg/ml). Aliquots (2 ml) of these solutions were transferred into test tubes with caps. After addition of 5% aqueous phenol solution (1 ml) and concentrated sulfuric acid (5 ml), the mixture was fully shaked, and then kept for 5 min at room temperature followed by 15 min incubation at 90 °C in a water bath. The absorbance of the resulting solution at 490 nm was read by an UV–vis spectrophotometer (UV-1800; Shimadzu, Kyoto, Japan) with distilled water as the blank. The obtained standard curve over a range of 0.01-0.05 mg/ml was Y = 5.542X + 0.083 with a squared regression coefficient (R^2^) of 0.992, where Y was the absorbance and X was the concentration of glucose. The content of TP was expressed as glucose equivalents.

### Extraction of total flavonoids

The supernatant of QYD after ethanol precipitation was evaporated under reduced pressure. The obtained dry sample was dissolved in deionized water, followed by adjustment of pH to 9.0 using 1.0 M NaOH solution. The solution was washed three times with chloroform, adjusted to pH 3.0 by 1.0 M HCl solution and loaded onto a polyamide resin (polycaprolactam type; Yoko, Wuhan, China) column. The column was flushed with 5 bed volumes of ionized water and 50% ethanol, with the latter eluate gathered. After removing the solvent, the final TF extract was obtained (Fig. 1).

The content of TF in the extract was determined by UV–vis spectrophotometry at 270 nm with icariin (Must Biotechnology, Chengdu, China) as the standard and methanol as the vehicle, as detailed in the entry ‘Yinyanghuo’ of Chinese Pharmacopeia (2015, volume 1). The obtained standard curve was Y = 36.709X + 0.018 (R^2^ = 0.998). In the equation, Y was the absorbance and X was the icariin concentration, and the linear range was 0.002-0.012 mg/ml. The TF content was expressed as icariin equivalents.

### Extraction of total saponins

Qian-Yu decoction was washed three times with petroleum ether to remove the non- and low-polar fractions, and then treated with water saturated n-butanol to extract saponins. The resulting organic phase was evaporated under reduced pressure and the crude saponins were obtained as a thick liquid. The sample was loaded into a macroporous adsorption resin (Type D101; SinoPharm Chemical Reagent, Shanghai, China) column via a dry-loading method. After washing the column with 5 bed volumes of deionized water and 30% ethanol, saponins were eluted using 5 bed volumes of 70% ethanol. The eluent was evaporated under low pressure to give the saponin extract (Fig. 1).

The TS content of the extract was tested by a vanillic aldehyde-perchloric acid colorimetric method with astragaloside IV (Must Biotechnology, Chengdu, China) as the standard [23]. The standard solutions of astragaloside IV in methanol at varying concentrations (0.25-1.00 mg/ml) were prepared. Aliquots (1.0 ml) of standard solutions were transferred into volumetric flasks (25 ml), and 0.5% vanillic aldehyde-acetic acid (1 ml) and perchloric acid (4 ml) were added. After shaking, the mixtures were heated at 60 °C for 10 min in a water bath, then cooled in an ice water bath, and finally diluted with methanol to a defined volume (25 ml). The absorbance of the resulting solutions at 554 nm was measured with sample-free solution as the blank. The standard curve was obtained as Y = 0.515X + 0.086 (R^2^ = 0.997), where Y was the absorbance and X was the astragaloside IV concentration. The linear range was 0.25-1.00 mg/ml. The content of TS was expressed as astragaloside IV equivalents.

### Animals

Male specific-pathogen-free (SPF)-bred Sprague–Dawley rats, aged eight weeks and weighed 180–220 g, were purchased from the Hubei Center for Diseases Control and Prevention (Wuhan, China). All rats were individually housed under SPF conditions at 22 ± 2 °C on a 12 h light/dark cycle, and fed using standard rat chow and water ad libitum. They were allowed one week of acclimatization prior to experiments. All animal protocols were approved by the Animal Ethics Committee of Wuhan Third Hospital, and conducted in uniformity with national guidelines of the care and use of laboratory animals.

### Acute toxicity study

In the preliminary examination, the median lethal dose (LD50) of DLP was not able to be found due to low toxicity. The maximum feasible dose (12.00 g/kg) was intragastrically administered to 20 male Sprague–Dawley rats, which were kept fasting and given water only for overnight before administration. The animals were observed to record mortality, toxic symptoms and body weight for 14 days after administration. At the 14th day, gross necropsies were performed on all animals.

### Induction of CP/CPPS in rats and treatment regimens

Rats were randomized into groups (10 rats per group): the normal group, the sham group, the model group, the positive control group and treatment groups, i.e., QYD, TP, TF, TS and any combination of extracts: polysaccharides-flavonoids-saponins (PFS), flavonoids-saponins (FS), polysaccharides-flavonoids (PF), polysaccharides-saponins (PS) combinations. The rats in the model, positive control and treatment groups were induced by carrageenan to CP/CPPS rats as described previously [24]. Briefly, both right and left ventral lobes of the prostate gland were injected with 0.1 ml sterile solution of 1.0% carrageenan. For the sham group, carrageenan solution was replaced by an equal volume of saline. All surgery was performed under isoflurane anesthesia (5% for induction and 3% for maintenance), and all efforts were made to minimize suffering. One week later, the rats in the treatment groups were intragastrically administered (1.0 ml/100 g) with DLP (3.00 g/kg), TP (482.3 mg/kg), TF (339.7 mg/kg), TS (380.2 mg/kg) and the extract combinations (e.g., PFS: (482.3 mg TP + 339.7 mg TF + 380.2 mg TS)/kg) once per day for three weeks, respectively. The dose of each extract was derived from DLP dose and the contents of the corresponding components in DLP and the extract. Cernilton (100.0 mg/kg) was given to the positive control group and saline to the normal, sham and model groups.

Body weight, food and water consumption by animals in each group were measured once each week. No death or abnormal behavior was observed during the course of the study. After final administration, the rats were starved for 12 h, weighed, and finally euthanized by carbon dioxide inhalation. The ventral prostates of rats were harvested and weighed instantly. Prostatic index was calculated as the ratio of prostate weight to body weight (mg/g). Each prostate was dissected into two halves, and one was fixed in 10% neutral buffered formalin for histological examination and the other was stored at −80 °C until use.

### Histological examination

After 24-h fixation, the tissues were gradually dehydrated in ethanol and embedded in paraffin, and then cut with a microtome into 5–6 μm thick paraffin sections. The sections were subjected to routine staining with hematoxylin and eosin (H&E) and examined under a light microscope.

### Measurements of TNF-α, IL-1β, COX-2 and PGE2

Frozen tissues were rinsed three times with ice cold saline. Known weights of the tissues were homogenized in cold 0.1 M Tris–HCl buffer (pH7.4) at 4 °C to give 10% homogenates (w/v), and centrifuged at 3000 × g for 20 min at 4 °C. The supernatants were stored at −20 °C until used. TNF-α, IL-1β, COX-2 and PGE2 in the supernatant were measured using commercial ELISA assay kits (TNF-α, IL-1β: Neobioscience, Shanghai, China; COX-2, PEG2: Cusabio, Wuhan, China), according to the manufacturers’ instructions. The assay tested each sample in triplicate. The results are expressed as pg/ml.

### Measurements of NO and iNOS

The measurements of NO and iNOS in the above homogenate supernatant were performed with nitrate/nitrite assay kits and enzymatic activity assay kits (Nanjing Jiancheng Bioengineering Institute, Nanjing, China), respectively, in line with the recommended procedures. Each sample was measured in triplicate. The level of NO is expressed as μmol/g, and the activity of iNOS is expressed as U/mg protein.

### Statistical analysis

All values are given as means ± standard deviations (S.D.). Statistical analysis was carried out using one-way analysis of variance (ANOVA) test, followed by Turkey’s post hoc test. *P* < 0.05 was considered statistically significant.

## Results

### Contents of TP, TF and TS

**Fig. 2:**
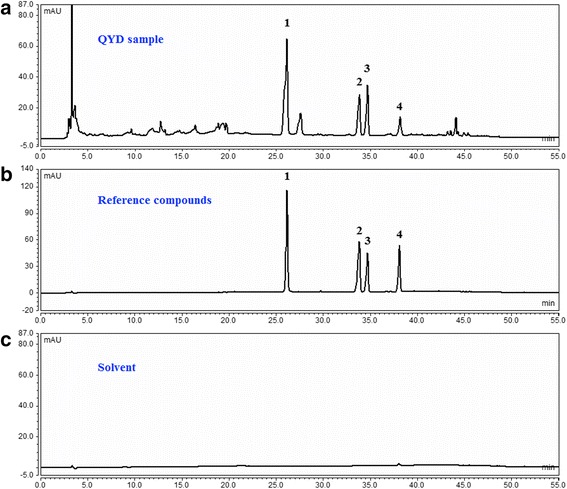
Fingerprint analysis of QYD using HPLC. **a** chromatogram of QYD lyophilized product (DLP); **b** chromatogram of standard references, (1) baicalin, (2) icariin, (3) formononetin and (4) wogonin; **c** chromatogram of the solvent (blank). The signals were monitored at 254 nm

Using spectrophotometric and colorimetric methods, the TP, TF and TS contents of DLP were found to be 11.3%, 6.2% and 8.2%. After TP, TF and TS were separately extracted from QYD, their contents in the extracts reached up to 69.0%, 63.4% and 53.6%, respectively. The HPLC fingerprint analysis of QYD was conducted, as shown in Fig. 2.

### Acute toxicity of Qian-Yu decoction

No mortality and no sign of toxicity were observed in 14 days after the administration of the maximum feasible dose (12.00 g/kg) of DLP. Gross necropsies indicated no apparent lesions in the major organs of animals. The doses up to 12.00 g/kg were considered as safe. Based on the results, the doses of 1.50, 3.00, 6.00 g/kg were selected for further evaluation of therapeutic activity on CP/CPPS.

### Prostatic index and histopathological analysis

**Fig. 3:**
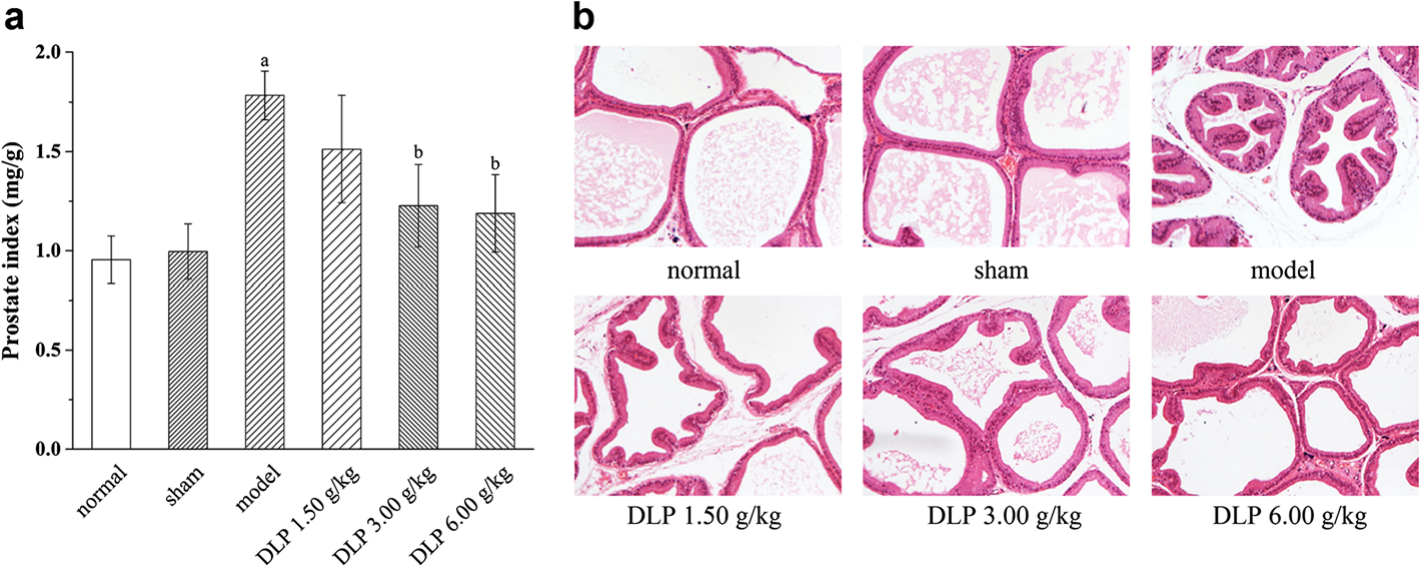
Prostatic indexes (**a**) and histopathological sections (**b**) of carrageenan-induced CP/CPPS rats after treatments of different doses (1.50, 3.00, 6.00 g/kg) of QYD lyophilized product (DLP). All the sections were stained with hematoxylin and eosin, and observed under light microscope at 100× original magnification. ^a^ *p* < 0.01when compared with the sham group; ^b^ *p* < 0.05 when compared to the model group

At first, we compared the effects of different doses of DLP (1.50, 3.00, 6.00 g/kg) on the PI levels and histopathology of CP/CPPS rats, as illustrated in Fig. 3. Obviously, the level of PI for the model group had a significant increase as compared with that for the sham group, while there was no significant difference between the sham and normal groups (Fig. 3a). The severity of condition in each group was histologically evaluated in terms of lymphocyte infiltration, morphological change of prostate acini and interstitial fibrosis (Fig. 3b). It can be seen that in contrast to the normal and sham groups without signs of inflammation, the model group was characterized by focal lymphocyte infiltration in the stroma, notably increased epithelial height, frequent luminal in-foldings, significant reduction of acinar diameter and enhanced interstitial proliferation. This confirmed the successful establishment of CP/CPPS model. Treatment with DLP at the doses of 1.50, 3.00, 6.00 g/kg significantly decreased PI levels and ameliorated the histopathological alternations of rat prostates. Moreover, DLP at the doses of 3.00, 6.00 g/kg were clearly more effective than that at the dose of 1.50 g/kg, and increasing the dose of DLP from 3.00 to 6.00 g/kg failed to improve its therapeutic activities. Therefore, the DLP dose of 3.00 g/kg was used for the decoction group in the following study.

**Fig. 4:**
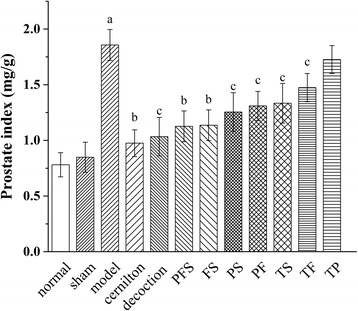
Prostatic indexes of carrageenan-induced CP/CPPS rats after three-week oral administration of QYD and its extracts. Decoction, rats treated with QYD lyophilized product (3.00 g/kg); TP, TF, TS, PFS, FS, PS and PF, rats treated with total polysaccharides (482.3 mg/kg), total flavonoids (339.7 mg/kg), total saponins (380.2 mg/kg) from QYD and their combined forms, i.e., polysaccharides-flavonoids-saponins, flavonoids-saponins, polysaccharides-saponins and polysaccharides-flavonoids combinations. All values are given as means ± S.D. ^a^ *p* < 0.01, when compared with the sham group; ^b^ *p* < 0.01, ^c^ *p* < 0.05 when compared with the model group

**Fig. 5:**
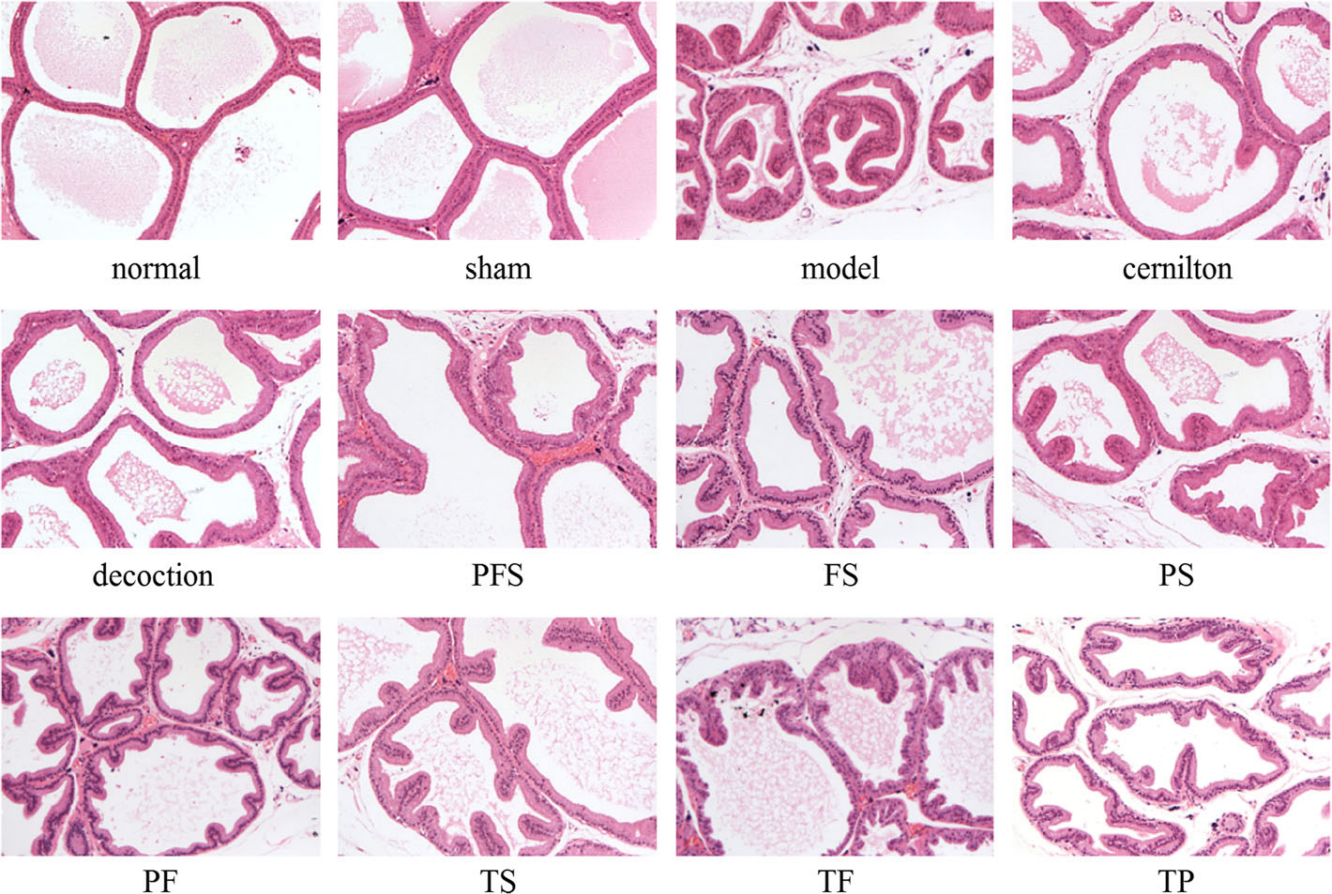
Histopathologic sections of the ventral prostates of carrageenan-induced CP/CPPS rats from different treatment groups. Decoction, rats treated with QYD lyophilized product (3.00 g/kg); TP, TF, TS, PFS, FS, PS, PF, rats treated with total polysaccharides (482.3 mg/kg), total flavonoids (339.7 mg/kg), total saponins (380.2 mg/kg) from QYD and their combined forms, i.e., polysaccharides-flavonoids-saponins, flavonoids-saponins, polysaccharides-saponins and polysaccharides-flavonoids combinations. Sections were stained with hematoxylin and eosin, and photographed at 100× original magnification

The effect of TP, TF, TS in QYD and their combined forms on the symptoms of CP/CPPS was further investigated, as shown in Figs. 4 and 5. The intragastric administration of TP, TF and TS reduced the PI level of CP/CPPS rats by 3.8%, 20.6% and 28.2%, respectively, while the PFS group showed the closest PI to the decoction group (Fig. 4). The PI level of the decoction group was in close proximity to that of the cernilton group. Also, Fig. 5 indicates that the histological changes of CP/CPPS rats were suppressed by the extracts and their combined therapies, and PFS had the most obvious effect. Treatment with QYD was very comparable to that with cernilton, resulting in a marked symptom amelioration.

### Effects of QYD and its extracts on TNF-α and IL-β

**Fig. 6:**
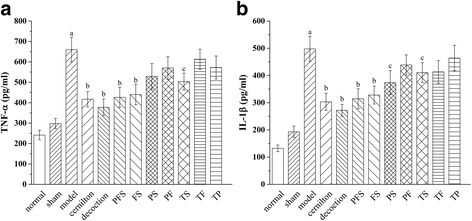
Effects of QYD and its extracts on prostatic TNF-α (**a**) and IL-β (**b**) levels of carrageenan-induced CP/CPPS rats. Decoction, rats treated with QYD lyophilized product (3.00 g/kg); TP, TF, TS, PFS, FS, PS and PF, rats treated with total polysaccharides (482.3 mg/kg), total flavonoids (339.7 mg/kg), total saponins (380.2 mg/kg) from QYD and all of their combined forms, i.e., polysaccharides-flavonoids-saponins, flavonoids-saponins, polysaccharides-saponins and polysaccharides-flavonoids combinations. Values are presented as means ± S.D. ^a^ *p* < 0.01, when compared with the sham group; ^b^ *p* < 0.01, ^c^ *p* < 0.05 when compared with the model group

The levels of TNF-α and IL-β in the rat ventral prostates of the model group were found to be much higher than those of the sham group (Fig. 6). The TNF-α and IL-β levels of the treatment groups exhibited varying decreases of 7.1% to 42.8% and 6.8% to 45.3% as compared with the model group, with those for the decoction group and the PFS group the lowest. The decoction group had lower levels of TNF-α and IL-β than the cernilton group, and the PFS group had no significant difference from the cernilton group.

### Effects of QYD and its extracts on COX-2, PGE2, iNOS and NO

**Fig. 7:**
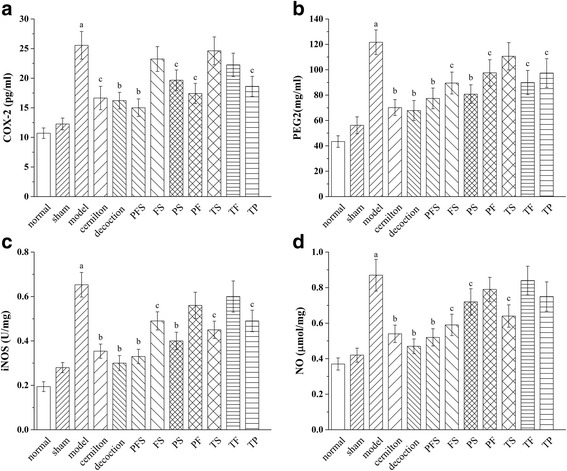
Effects of QYD and its preparative extracts on prostatic COX-2 (**a**), PGE2 (**b**), iNOS (﻿**c**﻿) and NO (**d**) levels of carrageenan-induced CP/CPPS rats. Decoction, rats treated with QYD lyophilized product; TP, TF, TS, PFS, FS, PS and PF, rats treated with total polysaccharides, total flavonoids, total saponins extracted from QYD and all of their combined forms, i.e., polysaccharides-flavonoids-saponins, flavonoids-saponins, polysaccharides-saponins and polysaccharides-flavonoids combinations. All values are given as means ± S.D. ^a^ *p* < 0.01, when compared with the sham group; ^b^ *p* < 0.01, ^c^ *p* < 0.05 when compared with the model group

As illustrated in Fig. 7, the levels of COX-2, PGE2, iNOS and NO in the rat ventral prostates of the model group were significantly upregulated when compared to the sham group, and the upregulation was attenuated by treatments with the test samples. The decoction group exhibited the lowest prostatic levels of PGE2, iNOS and NO among the treatment groups, while the COX-2 level of the PFS group was lower than those of the others. However, the effect of PFS on the production of these inflammatory mediators only had a small difference from that of QYD.

## Discussion

Qian-Yu decoction is a traditional herbal recipe consisting of five frequently used Chinese herbs, i.e., *Radix astragali*, *Herba epimedii*, *Herba leonuri*, *Cortex phellodendri* and *Radix achyranthis bidentatae*. As prescribed on clinical knowledge of traditional Chinese medicine, *radix astragali* and *herba epimedii* accounting for 60% of the total herbal weight were expected to function as the primary ingredients, and the rest assists in managing the condition. In this study, the therapeutic effect of QYD on carrageenan-induced CP/CPPS in rats was investigated. Injection of carrageenan led to increased PI level and abnormal prostatic histology in line with the previous observations [25, 26], as well as upregulation of inflammatory markers, which demonstrated a successfully established CP/CPPS model. Carrageenan is a highly sulfated polygalactan with 15-40% of ester-sulfate content and an average molecular weight well above 100 kDa [27]. Carrageenan has been widely used to induce inflammation in animal models and human cells [28, 29]. Carrageenan triggers inflammatory cascades in which Toll-like receptor 4 (TLR4) and B-cell leukemia/lymphoma 10 (BCL10) are critical [30, 31]. The inflammatory response initiated by carrageenan exposure activates both canonical, involving RelA (p65) and p50, and non-canonical, involving RelB and p52, pathways of nuclear factor kappa B (NF-κB) activation.

The treatment with QYD markedly decreased the level of PI and attenuated the histological changes of prostatic tissues. Moreover, the production of TNF-α and IL-β were significantly reduced in the decoction group, as was the case for COX-2, PGE2, iNOS and NO. The efficacy of QYD was highly comparable to that of cernilton, which is a recognized phototherapeutic agent of CP/CPSS. Therefore, QYD can be beneficial in treating CP/CPPS.

The extracts from QYD, TP, TF and TS, individually attenuated the symptom of CP/CPPS in rats, but obviously more weakly than QYD. Among them, TS suppressed the production of TNF-α, IL-1β, iNOS and NO more effectively, while the treatments with TP and TF resulted in relatively significant reduction in the levels of COX-2 and PGE2, respectively. The findings are in agreement with the previous works [32–37]. For instance, astragalus saponins inhibited the production of TNF-α, the overexpression of iNOS and the subsequent generation of NO in lipopolysaccharide (LPS)-stimulated mouse macrophage RAW264.7 [32]. In the same model, astragalus flavonoids reduced the release of TNF-α, IL-1β and NO in a dose-dependent manner [33]. Moreover, the presence of astragalus polysaccharides downregulated the expression of TNF-α and IL-1β in LPS-infected Caco2 cells and COX-2 expression in HepG2 cells [34, 35]. Guo *et al.* studied the protective effect of icariin, a major flavonoid of *herba epimedii*, on a rat model with brain dysfunction (inflammation) induced by LPS [36]. Their results showed that the expression of TNF-α, IL-1β and COX-2 in brain was significantly reduced by icariin. Also, icariin inhibited the mRNA expression of TNF-α, COX-2 and iNOS and further suppressed the secretion of TNF-α, PGE2 and NO in the lung of LPS-treated mice [37]. Recent studies suggested that the effects of the three extracts on modulating these inflammatory mediators might be more or less associated with inactivation of NF-κB [22, 37–41]. It has been well known that NF-κB activation upregulates gene expression of various inflammatory cytokines (e.g., TNF-α, IL-1β) and enzymes (e.g., COX-2, iNOS) [42, 43]. Meanwhile, TNF-α and IL-1β, as the key mediators of inflammatory response, also activate NF-κB and stimulate their own production and the production of other inflammatory mediators (including iNOS and COX-2) [44–46]. The upregulation of iNOS and COX-2 leads to increased synthesis of NO and PGE2.

Despite weak effects of individual extracts, the combined administration of three extracts demonstrated efficacy comparable to that of QYD while better than that of any combination of two extracts. Therefore, all three extracts contribute to the therapeutic effect of QYD on CP/CPPS, and constitute the major part of its effective components.

**Fig. 8:**
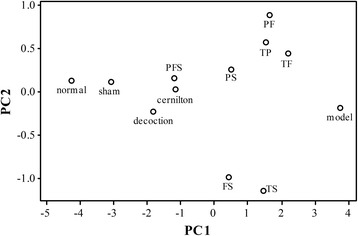
A plot of the scores of the second principal component (PC2) against the first principal component (PC1) of a principal component analysis on inflammatory markers. PC1 and PC2 contribute to 96.5% of the total variance in the data

A very useful way to compare the therapeutic effects of the test samples is to perform a principal component analysis (PCA) using all six markers of inflammation (TNF-α, IL-β, COX-2, PGE2, iNOS and NO) as variables. PCA can convert a set of possibly correlated variables into a set of linearly uncorrelated variables called principal components (PCs) that contain exactly the same information. The first few PCs usually contain the vast majority of the information in the data so that the dimensionality of the data can be reduced. In the present case, the first two PCs accounted for 96.5% of the total variance in our data. A plot of the scores of the second PC versus the first PC revealed how close the inflammatory states of the animal groups were in terms of the distance between the points in the two-dimensional plot, see Fig. 8. It can be easily seen that the point for the decoction group is closer to the sham group than the cernilton group, and the PFS group is quite close to the sham and decoction groups. Very interestingly, the TP, TF and TS groups stay far away from the sham group in similar distances, while the TS group is distant from the TP and TF groups, which stay close to each other. This reveals that the effects of TS on CP/CPPS are rather different from those of TF and TP. Thus, there is a cooperative effect among these three extracts involved in the therapeutic mechanism of QYD [47, 48].

## Conclusion

In the present study, the therapeutic activity of QYD and its three preparative extracts (i.e., TP, TF and TS) against CP/CPPS was investigated on carrageenan-induced rat models. The results showed that QYD had a potent therapeutic activity on CP/CPPS, which was even slightly stronger than the popular plant extract, cernilton. The individual extracts exerted a weak efficacy at the same doses as contained in a dose of decoction, whereas the combined therapy with the three extracts produced a comparable effect to the decoction. Hence, QYD may be considered as an effective treatment option for CP/CPPS, and polysaccharides, flavonoids and saponins make up its major effective components. Note that the components we discussed in the decoction might differ from the original components of the herbs in the prescription, because of unavoidable chemical changes when decocting [49]. In the future, we will do more work to identify the principal individual effective components of QYD.

## Abbreviations

BCL10: B-cell leukemia/lymphoma 10
COX-2: Cyclooxygenase-2
CP/CPPS: Chronic prostatitis/chronic pelvic pain syndrome
DLP: Lyophilized product of Qian-Yu decoction
FS: Flavonoids-saponins combination
IL-1β: Interleukin-1beta
iNOS: Inducible nitric oxide synthase
NO: Nitric oxide
PC: Principal component
PCA: Principal component analysis
PF: Polysaccharides-flavonoids combination
PFS: Polysaccharides-flavonoids-saponins combination
PGE2: Prostaglandin E2
PI: Prostatic index
PS: Polysaccharides-saponins combination
QYD: Qian-Yu decoction
TF: Total flavonoids
TLR4: Toll-like receptor 4
TNF-α: Tumor necrosis factor alpha
TP: Total polysaccharides
TS: Total saponins
